# Efficacy of a Novel ACE-Inhibitory Peptide from *Sargassum maclurei* in Hypertension and Reduction of Intracellular Endothelin-1

**DOI:** 10.3390/nu12030653

**Published:** 2020-02-28

**Authors:** Yajun Zheng, Yufeng Zhang, Sang San

**Affiliations:** 1 Food Science Institute of Shanxi Normal University, Linfen 041004, China; 2 Coconut Research Institute of Chinese Tropical Agriculture Academic, Haikou 570100, China; zhangyufeng2017@yeah.net; 3 Yunnan Institute of Food Safety, Kunming University of Science and Technology, Kunming 650093, China; zhangbaoshan2015@yeah.net

**Keywords:** *Sargassum maclurei* protein, angiotensin I-converting enzyme, in silico analysis, inhibition kinetics, molecular docking, spontaneously hypertensive rats, endothelin-1

## Abstract

*Sargassum maclurei* is a potential protein resource because of its high protein content and relatively balanced amino acid composition. To promote its usage in food, medical, or other industries, *S. maclurei* protein was hydrolyzed by pepsin and papain to obtain bioactive peptides. The *S. maclurei* protein hydrolysates (SMPHs) were purified using gel chromatography and reversed-phase high performance liquid chromatography (RP-HPLC), and 12 major fractions were obtained. The fraction D11 with the highest angiotensin I-converting enzyme (ACE) inhibition (61.59%, at 1 mg/mL) was subjected to liquid chromatography-mass spectrometry (LC-MS/MS) analysis, and about 17 peptides were identified, of which the RWDISQPY (1063.5 Da) was chosen to be synthesized based on in silico analysis. The RWDISQPY demonstrated high ACE inhibition ability (IC_50_: 72.24 μM) with competitive inhibition mode, and could effectively (*p* < 0.05) lower the systolic blood pressure and diastolic pressure of spontaneously hypertensive rats at the concentration of 150 mg/kg body weight. The results of the molecular docking simulation demonstrated that RWDISQPY could bind with the active sites S1 and S2 of ACE via short hydrogen bonds. Moreover, RWDISQPY showed acceptable endothelin-1 suppressing capacity (26.21% at 1.5 mg/mL). These results indicate that *S. maclurei* could be developed into functional foods such as antihypertensive products.

## 1. Introduction

The important role of angiotensin I-converting enzyme (ACE) in increasing blood pressure has been demonstrated in the past decades, as about 9.4 million patients die from hypertension worldwide every year and 1.6 billion people are suffering from high blood pressure [1]. Reducing blood pressure to normal levels is crucial, and thus more attention has been focused on the effects of ACE-inhibitory substances. Compared to synthetic drugs, the use of ACE-inhibitory peptides derived from food sources is a more effective way to reduce blood pressure without unacceptable side effects [2]. More importantly, undesirable dietary habits are the first risk factor responsible for the development of hypertension and diet-related cardiovascular disease. In China, the age-standardized rate of diet-related cardiovascular disease deaths was 299 deaths per 100,000 in 2017, while Pakistan had the highest proportions of diet related cardiovascular disease deaths and disability-adjusted life-years (62% of deaths and 66% disability, respectively) [3]. Thus, the control and improvement of diet is the most important and economical option to achieve a desirable blood pressure-lowering effect [4]. In addition, numerous studies have revealed that the excessive expression of endothelin-1 (ET-1) in the endothelium makes a positive contribution to hypertension and atherosclerosis, which are the major precursors of cardiovascular diseases [5]. Thus, inhibition of endothelin-1 activity is also a target of antihypertensive substances. Throughout recent years, many studies have investigated the identification, characterization, and in vivo antihypertension of ACE-inhibitory peptides derived from various food resources. Some of them have focused on the bioavailability and action mechanism of these peptides [4,6,7,8]. Structure–activity relationship studies have demonstrated that the ACE inhibition activity of peptides is mainly dependent on the connection mode between the C-terminal residues of the peptides and the ACE active sites [9]. Moreover, ACE-inhibitory peptides need to remain as relatively intact active peptide chains when they are absorbed by intestinal epithelial cells and enter into the blood circulation to play a blood pressure reducing role [10,11]. Thus, for a new ACE-inhibitory peptide, it is necessary to investigate the in vivo bioactivity, and interaction between ACE and the peptides.

Marine products such as fish, alga, and shellfish are high quality medical and edible protein resources. In recent years, numerous antihypertensive peptides have been identified in marine product proteins [4,5,6,10]. *Sargassum maclurei* (S. *maclurei Setch*), a type of brown seaweed, is grown mainly in tropical coastal areas off the south of China, especially in the coastland of Guangdong Province, Guangxi Province and Hainan Island. *Sargassum* is the main source of algin in China. It is also used as feed and green manure, but not usually used as food [12]. In recent years, many health benefits of a diet supplemented by *S. maclurei* have been reported, such as a low incidence of cancer, hypertension and goiter [13]. In China and Korea, *S. maclurei* is used to treat some diseases like chronic bronchitis, hypertension, skin diseases, esophagitis, and liver organ swelling [14,15]. Moreover, it has been demonstrated that *S. maclurei* or its extraction has some important other bioactivities, including antitumor, anti-inflammatory, antimicrobial, hypoglycemic and antioxidant activity. *S. maclurei* is a potential protein resource for its high yield (about 800,000 tons per year in China), high protein content (14.1–19.1 g/100 g) and relatively balanced amino acid composition [12]. However, studies referring to *S. maclurei* have been focused on polysaccharide, phlorotannins, fucoidan and polyphones [16,17,18,19,20]. Data in the literature on the protein and bioactive peptides of *S. maclurei* are limited [21,22,23,24]. The pre-experiment of this study demonstrated that *S. maclurei* protein could be extracted with a diluted acidic solution. After hydrolysis with pepsin, the *S. maclurei* protein hydrolysates (SMPHs) showed considerable ACE inhibition activity (43.67%, at 1.0 mg/mL), indicating that antihypertensive peptides may be obtained from it. Therefore, peptides with ACE inhibition activity were identified from SMPHs in the current study. A molecular docking simulation was employed to study the structure–activity relationship. The ACE inhibition kinetics, in vivo antihypertension and effects on intracellular ET-1 were also investigated to determine the potential antihypertensive mechanism.

## 2. Materials and Methods

### 2.1. Materials

*Sargassum maclurei*, which was picked in the intertidal zone in May 2017, was obtained from Hainan Weizao Limited *Co*., Sanya, Hainan, China. ACE (from rabbit lungs) was purchased from Sigma-Aldrich Co. (Louis, MO, USA). EA.hy926 cells were obtained from the Type Culture Collection of the Chinese Academy of Sciences, Shanghai, China. Sephadex G-15 was purchased from Yuanye Biotechnology Co., Ltd., Shanghai, China. Human endothelin-1 Elisa kit, DMEM (Dulbecco’s modified Eagle’s medium), and MTT (3-(4,5-dimethyl-2-yl)-2,5-diphenyltetrazolium bromide) were obtained from Solarbio (Beijing, China). Pepsin (5.0 × 10^4^ U) and papain (2.0 × 10^5^ U) were from Supei Biotech Co. (Shanghai, China). All other reagents used were of analytical grade.

### 2.2. Enzymatic Hydrolysis of Sargassum maclurei Protein

The *S. maclurei* was washed with distilled water three times and cut into pieces with a diameter of about 1 cm [22]. The pieces were dried at 50 °C for 8 h in an oven (101-D, Hengxin Electronic Ltd., Hebi, Henan, China), and then milled and passed through a 60 mesh sieve. Ten grams of the dried *S. maclurei* powder was mixed with 250 mL of 0.1 M HCl. After treatment with ultrasound (100 W, 35 °C) for 20 min, the mixture was gently stirred at 45 °C for 3 h and then filtered, and the filtrate was collected. The residue was again mixed with 0.1 M HCl, and the extraction was repeated in triplicate. The filtrate was pooled and centrifuged at 10,000 *g* for 20 min. The supernatant was collected, dialyzed against distilled water for 24 h, and freeze-dried to obtain *S. maclurei* protein. An automatic amino acid analyzer (HITACHI L-8900, Tokyo, Japan) was used to determine the amino acid composition according to the procedure described by Wang et al. [25].

Pepsin (80 U/g) was added into *S. maclurei* protein suspensions (3 g/100 mL, dispersed in 0.2 M acetate buffer). The mixture was adjusted to pH 2.0 and placed at 37 °C for 2 h, and then adjusted to pH 7.0. Then papain (60 U/g) was added and incubated at 50 °C for another 3 h. After that, the reaction solution was incubated at 100 °C for 10 min to stop the hydrolysis, and then centrifuged at 10,000 *g* for 15 min. The supernatant was pooled and freeze-dried to obtain *S. maclurei* protein hydrolysates (SMPHs). The trinitrobenzenesulfonic acid method [26] was used to determine the hydrolysis degree of the SMPHs.

### 2.3. ACE Inhibition Activity and Inhibition Kinetics

Briefly, the reaction system comprised 50 µL ACE (25 mU), 150 µL of 8.3 mM HHL (N-hippuryl-His-Leu hydrate) and 50 µL of sample solutions [27]. After incubation in a shaking water bath (37 °C) for 60 min, the reaction was terminated by addition of 1 M HCl (250 µL). The hippuric acid formed was subsequently extracted by ethyl acetate (1.4 mL). After centrifugation, 1 mL of the supernatant was collected and evaporated at 80 °C for 60 min in a vacuum. The residue was mixed thoroughly with 2 mL of distilled water and measured at 228 nm with a spectrophotometer (UV-1930PC, Meixi Instrument Co., Ltd., Huizhou, Guangdong, China). The inhibitory activity was calculated by comparing the absorbance of ACE solutions with and without inhibitors at 228 nm. The IC_50_ value was the concentration of inhibitor needed to inhibit half of the activity of ACE. The ACE inhibition kinetics of samples was analyzed by Lineweaver–Burk plots of 1/V versus 1/HHL in the presence of the inhibitor [28]. The concentrations of HHL used were 0.76 to 7.60 mM.

### 2.4. Purification by Gel Chromatography and RP-HPLC

SMPHs (2 mg/mL) were filtered through a 0.22 µm filter and subjected to gel chromatography separation [29]. The gel chromatography was performed on a Sephadex G-15 column (Φ 1.2 × 100 cm) with 0.1 M HCl as eluate (2.8 mL/min) and monitored at 220 nm. The fractions were pooled, freeze-dried, and their ACE-inhibitory activity was determined, after which the fraction that demonstrated the highest activity was chosen for further separation by semi-preparative RP-HPLC. The RP-HPLC purification was carried out with a Zorbax C_18_ column (SB-300, Φ 9.4 × 250 mm, Agilent Technologies, Palo Alto, CA, USA) using distilled water (containing 0.1% TFA) as mobile phase A and a linear gradient of acetonitrile (5%–35%, in 30 min) as phase B. The flow rate was 2.2 mL/min and the monitoring wavelength was 220 nm. Each chromatographic run was repeated 15–20 times and the subfractions were pooled and freeze-dried. The subfraction with the highest activity was subjected to peptide sequence analysis.

### 2.5. Peptide Sequence Identification

Peptide identification was performed using LC–MS/MS coupled to an Eksigent Nano LC (Eksigent Technologies, Dublin, CA, USA) and Thermo LTQ linear ion trap mass spectrometer (Thermo Fisher, San Jose, CA, USA) [30]. Briefly, the nano-LC separation was carried out with 2% acetonitrile (containing 0.1% formic acids) as mobile phase A and 80% acetonitrile (containing 0.1% formic acids) as mobile phase B. Gradient elution was performed following three steps: 0–85 min, the phase B linearly increased from 5% to 50%; 86–95 min, phase B was increased from 85% to 95%; 96–125 min, phase B was kept at 95%. The peptide sequence was analyzed by MS/MS with a capillary temperature of 200 °C, normalized collision energy of 35%, and spray voltage of 2.2 kV at a scan range of 300–2000 *m/z*. Based on the acquired MS/MS, Proteome Discoverer 2.1 (Thermo Fisher Scientific) and Xcalibur software (version 2.0.7, Thermo Fisher Scientific, Les Ulis, France) were used to interpret the peptide sequences.

### 2.6. In silico Screening of Peptides and Chemical Synthesis

Following the method of Piovesana et al. [31], the theoretical ACE-inhibitory activity and antihypertensive (AHT) ability of the peptides obtained by LC–MS/MS analysis were screened using the AHTPDB databases (http://crdd.osdd.net/raghava/ahtpdb/) and BIOPEP (http://www.uwm.edu.pl/biochemia/index.php/en/biopep). The potential ACE-inhibitory peptides were chose based on the B value (the potential biological activity calculated using the activity of the sequence motif published in BIOPEP database) and average local confidence (ALC; if it was above 85%). Moreover, the potential antihypertensive (AHT) ability was calculated based on the vector machine software score (SVMS; greater than zero). In addition, the peptides’ physicochemical properties were also predicted, including the hydrophobicity, net hydrogen and calculated isoelectric point (PI). Assisted with the in silico analyses, peptide sequences predicted to have ACE-inhibitory activity or antihypertensive activity were further synthesized by the standard solid phase method in Yaoqiang Biotech Limited Co. (Shanghai, China). The synthesized peptide was higher than 98% pure and verified by RP-HPLC.

### 2.7. Molecular Docking

The Build Protein function was used to construct the peptide structure and molecular modeling analysis was studied with the SYBYL-X 2.1.1 software [9]. The crystal structure of ACE (PDB: 1O8A) was downloaded from the Protein Data Bank (http://www.rcsb.org/pdb/home/home.do). The Dock Ligands function was used to form one pocket from 1O8A, into which the peptides were docked by molecular visualization [32]. It was necessary to remove all of the water molecules and unwanted substructures before the docking, and essential polar hydrogen atoms were added. The docking conformations between the purified peptides and the ACE active site were judged mainly by relying on the T-scores, which indicate the degree to which the inhibitors bind to ACE. Moreover, the hydrogen bond and distance were also calculated.

### 2.8. Antihypertensive Effect In Vivo

Twenty male spontaneously hypertensive rats (SHRs; 11 weeks, 240 ± 20 g body weight) from Vital River Laboratory Animal Technology Co., Ltd. (Beijing, China) were randomly divided into five groups of four rats each: negative control, positive control, and high-, middle- and low- dosage groups. Normal diet and tap water were freely available. Rats in the high-, middle-, and low-dose groups were orally given the synthetic peptide (RWDISQPY) via gastric intubation at 150, 100, and 50 mg/kg/body weight once daily, respectively. Meanwhile, rats in the positive control were orally given captopril (14 mg/kg/body weight once daily), and rats in the negative control were just administrated saline (0.9%). The diastolic blood pressure (DBP) and systolic blood pressure (SBP) of the rats were measured by the tail-cuff method once a week [33]. The heart rate was recorded simultaneously. The animal experiment was conducted in conformity with institutional guidelines for the care and use of laboratory animals in Shanxi Normal University (No. 20181003, 16 September 2018), Linfen, China. All of the rats received human care conforming to the National Institutes of Health Guide for Care and Use of Laboratory Animals (National Institute of Health Publication No. 85–23, revised).

### 2.9. Effects on Intracellular Endothelin-1 (ET-1)

In a humidified atmosphere with 5% CO_2_, the EA.hy926 cells (a permanent hybridoma of the human umbilical vein endothelial cells and human lung carcinoma cell line A549) grown in 96-well plates were cultured in DMEM containing non-essential amino acids (10 mg/mL), streptomycin (0.1 mg/mL), and fetal bovine serum (0.1 g/mL) at 37 °C [5]. The cells (1.0 × 10^5^ cells mL^−1^) were treated with the synthesized peptides (0.5–1.5 mg/mL) for 24 h, and then the MTT assay [34] was used to measure the cytotoxicity levels of the peptides. Moreover, EA.hy926 cells seeded in another 96-well plate (1.0 × 10^5^ cells mL^−1^) were cultured in DMEM at 37 °C for 24 h, and then treated with synthesized peptides (1–2 mg/mL) for 48 h. After that, the ET-1 content of the growth medium in each well was quantified by the ET-1 Elisa kit according to the instructions. Cells in the blank group were not treated with samples, while the cells in the positive group were treated with captopril (1 mg/mL). The inhibition ability on ET-1 was calculated as follows:

(1) $$\mathrm{Inhibition}\;\mathrm{abliliy}\;\left(\%\right)=\left(C_{B}-C_{s}\right)/C_{B}\times 100\%$$

where C_B_ was the ET-1 content of the growth medium in the blank group and C_S_ was the ET-1 content of the growth medium treated with the samples.

### 2.10. Statistical Analysis

Results are expressed as the mean ± standard deviation (*n* ≥ 3), and Duncan’s multiple range test was used for variance analysis (*p* < 0.05).

## 3. Results and Discussion

### 3.1. Preparation of SMPH

The extraction ratio of *S. maclurei* protein by 0.1 M HCl was 8.47 g/100 g dry *sargassum* in this study. Moreover, the amino acid composition is shown in Table 1. The results demonstrate that *S. maclurei* protein is rich in Glu, Asp and Ala. The essential amino acids accounted for 29.78 g/100 g protein, which is higher than the recommend value by FAO/WHO (Food and Agricultural Organization/World Health Organization) [35]. This result suggests that *S. maclurei* is a good potential protein resource and could be developed as a protein supplement [24,36]. Additionally, the aromatic amino acid content was 6.14 g/100 g protein, and the content of hydrophobic amino acids was 27.88 g/100 g protein. It was reported that these two kinds of amino acids mainly contribute to the ACE-inhibitory activity of proteins or peptides [4]. After being subjected to hydrolysis by pepsin and papain, the hydrolysis degree of the SMPHs was 16.82% ± 2.31%, which was lower (*p* < 0.05) than that of tilapia skin gelatin hydrolysates and Pacific cod skin gelatin hydrolysates prepared by the same enzymes [2,9]. Moreover, the ACE inhibitor activity of the SMPHs was 43.67% ± 4.03%.

### 3.2. Purification of the ACE-Inhibitory Peptides

**Figure 1:**
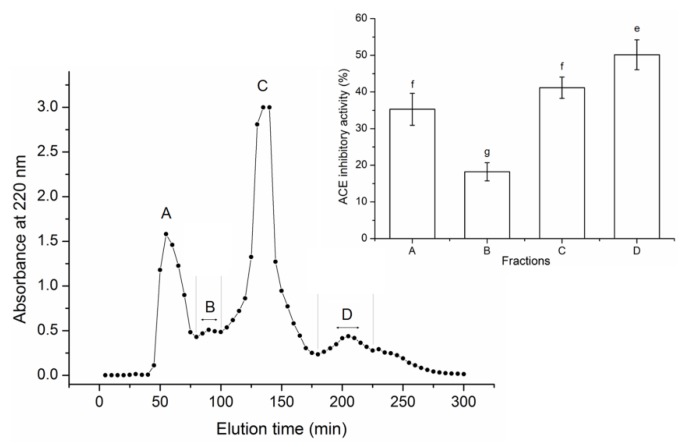
Purification profiles of *sargassum maclurei* protein hydrolysates (SMPHs) on Sephadex G-15 gel chromatography and the angiotensin I-converting enzyme (ACE) inhibition capacity of the subfractions. Uppercase letters (A–D) above the line represent the subfractions. Different lowercase letters (e–g) on the bars indicate a significant difference (*p* < 0.05).

As shown in Figure 1, after purification with Sephadex G-15 gel, the SMPHs were separated into four major peaks, named fraction A to D. Fraction D showed the highest activity (50.14% ± 4.07%, at 1.0 mg/mL). Thus, it was applied to RP-HPLC separation with a semi-preparative column.

**Figure 2:**
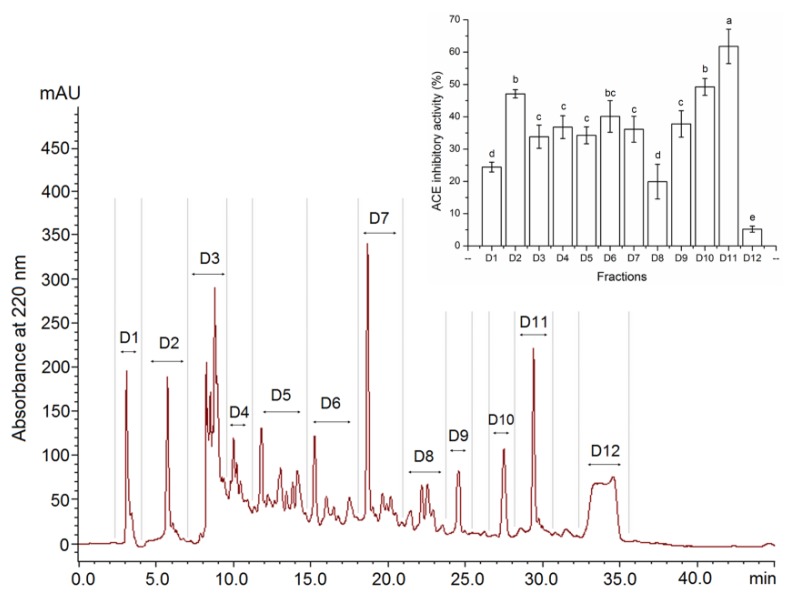
Semi-preparative RP-HPLC profiles of fraction D and ACE inhibition activity of each fraction. Different lowercase letters (e–g) above the bars indicate significant differences (*p* < 0.05).

As shown in Figure 2, there were 12 subfractions (D1 to D12) that appeared in the elution profile of fraction D using the semi-preparative RP-HPLC. These fractions were collected and ACE-inhibitory activity was measured. Among them, fraction D11, possessing the highest activity (61.79% ± 5.33%, at 1.0 mg/mL) was chosen for peptide sequence analysis. The extraction rate of D11 was 0.94 g/100 g dry *S. maclurei*.

### 3.3. In Silico Screening and Chemical Synthesis of ACE-Inhibitory Peptides

The results in Table 2 demonstrate that there were 17 peptides identified in fraction D11 by LC–MS/MS analysis. The potential antihypertensive peptides were screened based on the theoretical ACE-inhibitory activity (B) and AHT prediction, which were mainly dependent on ALC (average local confidence; > 85%) and SVMS (vector machine software score, > 0) of the peptides calculated using the BIOPEP and AHTPDB databases, respectively [31,37]. The potential ACE-inhibitory activity, antihypertensive activity and physicochemical properties of the 17 peptides are shown in Table 2. Among the 17 peptides identified in the SMPHs, only the peptide RWDISQPY (1063.5 Da) showed potential ACE-inhibitory activity and antihypertensive ability, which was because of the calculated B value (0.0016), high score of ALC value (91%), and SVM (0.34) [37]. Thus, it was chosen for chemical synthesis. The certification of the synthesized peptide by HPLC and LC-MS/MS is shown in Figure S1 and Figure S2.

**Figure 3:**
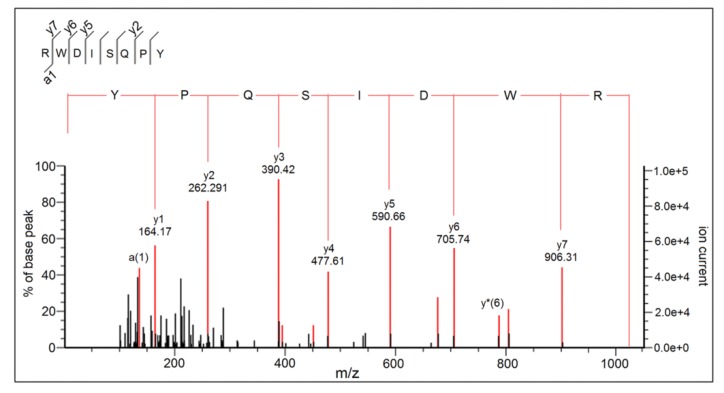
ESI-MS/MS spectrum of RWDISQPY identified in kernel expeller glutelin-2 hydrolysates.

**Figure 4:**
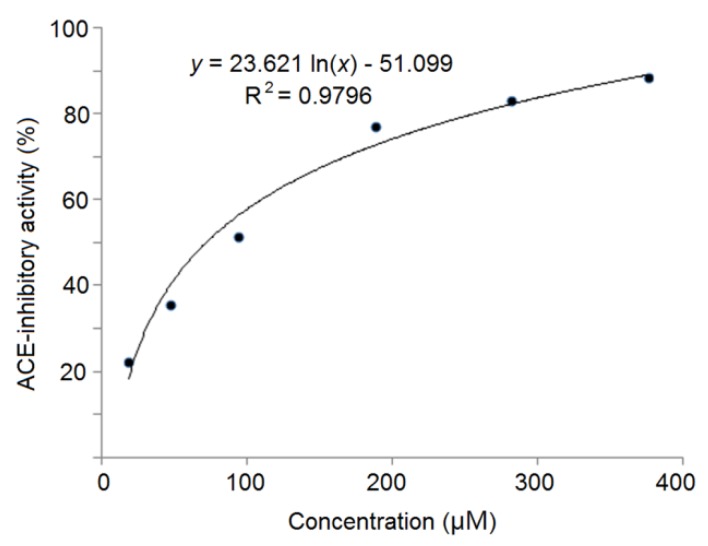
The ACE-inhibitory activity and the regression analysis of RWDISQPY.

The mass spectra by Nano-LC–ESI–MS/MS of RWDISQPY are shown in Figure 3. The relationship between the ACE inhibition capacity (*y*) and the concentrations of RWDISQPY (*x*) is shown in Figure 4. The results in Figure 4 demonstrate that the regression equations of RWDISQPY are *y* = 21.526 ln(*x*) − 47.294 (R^2^ = 0.9696), from which the IC_50_ value of RWDISQPY was calculated to be 72.24 μM. The IC_50_ value of RWDISQPY was lower than that of the peptides VSRHFASYAN (210 μM) identified in *Stichopus horrens* and FGMPLDR (219.35 μM) isolated from the marine macroalga *Ulva intestinalis* [8,38]; but higher than that of peptides QAGLSPVR (63.85 μM) from tilapia skin gelatin, FAS (0.47 μM) found in Antarctic krill and the ACE inhibitor drug captopril (0.023 μM) [5,9]. The results also demonstrate that RWDISQPY has a relatively high inhibition activity on ACE.

Now, it has been demonstrated that hydrogen bonds formed between the ACE active sites (S1, S1′, and S2) and peptides’ C-terminal tripeptide are crucial to ACE inhibition [6]. Previous studies found that peptides with hydrophobic amino acid residues, especially aromatic residues, and/or Pro in C-terminal tripeptides were more easily bonded to the active sites of ACE [8,39]. With respect to the peptides identified in SMPH, RWDISQPY contained Tyr and Pro at the C-terminal tripeptide. Moreover, the content of hydrophobic amino acid residues was 50.00% (Table 2). These structural features all significantly contributed to its relatively high ACE-inhibitory activity (72.24 μM), which was higher (*p* < 0.05) than that of the peptides identified from horrens protein (GSAGY, IC_50_: 706 μM) and casein (NMAINPSKENLCSTFCK, IC_50_: 129.07 μM) [38,40]. Furthermore, the Arg at the N-terminal of RWDISQPY contributed substantially to the high activity [41]. An increasing number of studies have found that, apart from molecular mass and C-terminal tripeptides, other amino acid sequences such as the N-terminal residue, C-terminal tetrapeptide, pentapeptide, and even heptapeptide of peptides also contribute to the inhibitory activity [25,41].

### 3.4. Inhibition Kinetics of Synthetic Peptides

**Figure 5:**
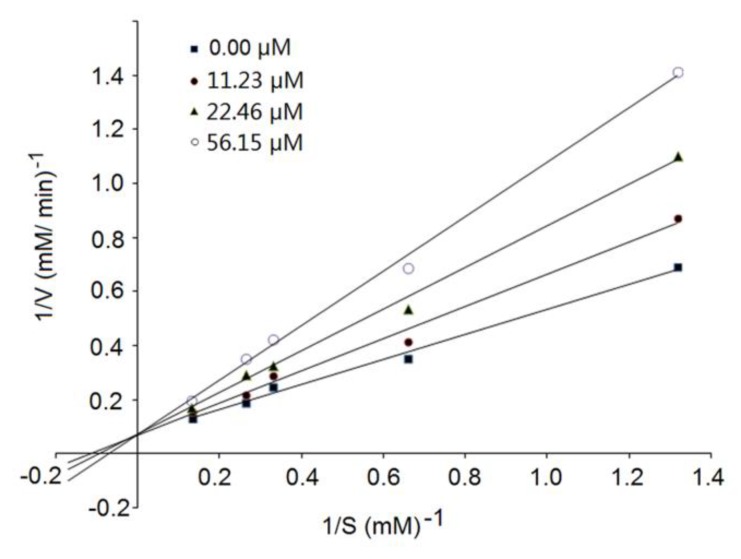
The Lineweaver–Burk plots of the ACE inhibition of RWDISQPY.

The kinetic constants of ACE as shown in Figure 5 reveal that the Michaelis–Menten constant (*K_m_*) of the reaction decreased while the maximum velocity (*V_max_*) remain unchanged with an increasing concentration of RWDISQPY, which was the characteristic of competitive inhibition modalities. This result suggests that RWDISQPY can competitively interact with the active site of ACE, and hinder ACE binding to substrates (like angiotensin-1 and bradykinin) [28]. Compared to noncompetitive and uncompetitive inhibitors, competitive peptides seem to exhibit relatively higher ACE-inhibitory activity [4]. Bhaskar et al. [41] and Lin et al. [28] also identified peptides TVGMTAKF, QLLLQQ and PFPGPIPN with competitive inhibition patterns.

### 3.5. Molecular Docking Simulation

A molecular docking study is an effective way to observe the molecular interactions between ACE-inhibitory peptides and ACE active sites, contributing to understanding the structure–activity relationship of bioactive peptides.

**Figure 6:**
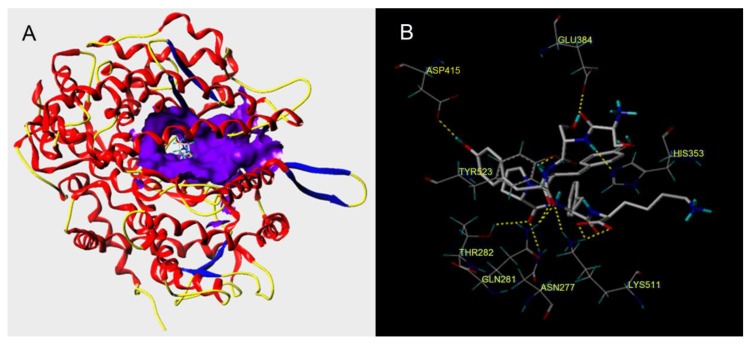
General overview (**A**) and local overview (**B**) of the best-ranked docking pose of RWDISQPY binding with ACE (PDB: 1O8A).

As shown in Figure 6 and Table 3, the docking simulation of the ACE–ligand complexes was well-performed between ACE and RWDISQPY. After docking, the T-Score of RWDISQPY was 10.70, indicating a strong binding power of the peptides and ACE [9]. Moreover, it was demonstrated that the hydrogen bond interaction force is very important to the stability of the docking complex [6].

The results in Table 3 demonstrate that there were 10 hydrogen bonds between ACE residues and RWDISQPY. Moreover, the distances of these hydrogen bonds to the ACE residues were very short, suggesting that the RWDISQPY was strongly bonded to the enzyme. These data suggest that RWDISQPY can interact effectively with ACE, mainly attributable to its high ACE-inhibitory activity (Table 2 and Figure 4). Moreover, the results in Figure 6b demonstrate that RWDISQPY can form 10 hydrogen bonds with eight amino acid residues of ACE, including Glu384, Asp358, His353, Tyr523, Thr282, Gln281, Asn277 and Lys511. More importantly, the residues Glu384 and Tyr523 belong to the ACE active site S1, and the residues His353, Gln282 and Lys511 are part of the ACE active site S2 [42]. This result reveals that RWDISQPY can inhibit ACE activity through interacting with the key active sites S1 and S2 of ACE, corresponding to its competitive inhibition modalities (Figure 4). Wang et al. [25] also found that peptide YSK bonding with the key residues Ala354, Gln281 and His353 is competitive.

### 3.6. Antihypertensive Effect of Peptides

**Figure 7:**
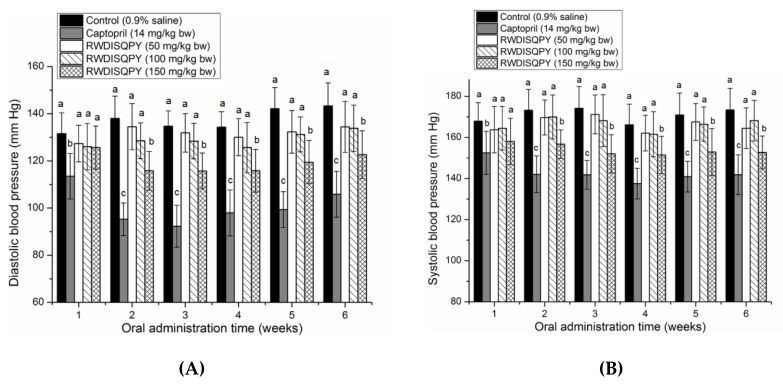
Effects of oral administration of RWDISQPY on diastolic blood pressure (**A**) and systolic blood pressure (**B**) of spontaneous hypertensive rats (SHR). SHRs in the low-, middle- and high-dose groups were orally administered peptides at 50, 100 and 150 mg/kg body weight (bw) every day, respectively. SHRs of the positive control group were given captopril at 14 mg/kg body weight once daily, whereas the SHRs in the blank group were just given saline (0.5 mL). Different small letters above the bars (a–c) indicate significant differences (*p* < 0.05).

As shown in Figure 7, oral administration of RWDISQPY at a high dose of 150 mg/kg body weight could significantly (*p* < 0.05) reduce both the DBP and SBP of SHRs from the second week, although the effect was weaker than that of captopril (*p* < 0.05). Moreover, the lowering effect of RWDISQPY on DBP and SBP showed an obvious dose-dependent relationship throughout the experiment period. The results suggest that RWDISQPY has an antihypertensive effect on SHRs, which is in accordance with the predicted result by the AHTPDB databases (Table 1). In addition, the results in Figure S3 demonstrate that administration of RWDISQPY had no side effect on heart rate and normal increase in body weight of SHRs.

### 3.7. Effects on Intracellular Endothelin-1 (ET-1)

**Figure 8:**
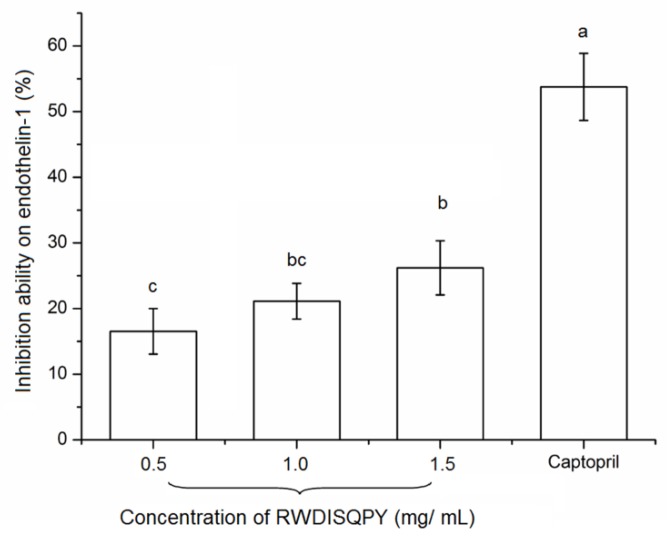
Effects of peptide RWDISQPY on intracellular endothelin-1 with captopril (1.0 mg/mL) as a comparison. Different small letters above the bars (a–c) indicate significant differences (*p* < 0.05).

Excessive expression of ET-1 is one of the endogenous mediators of cardiovascular disorders, such as atherosclerosis and hypertension [5]. RWDISQPY at different concentrations all exhibited considerable suppression ability (16.54%–26.20%) on ET-1 production (Figure 8). This result means that RWDISQPY may provide an antihypertensive effect through affecting the ET-1 and nitric oxide system, which is one of the most important systems involved in regulating blood pressure [5]. Moreover, it was reported that the endothelin converting enzyme (ECE) and protein kinase C (PKC) catalyzed the conversion of big endothelin into ET-1. Some factors like epinephrine, ACE, insulin and oxidative or physical damage on vascular endothelin cells could promote the production of ET-1, while nitric oxide, atrial natriuretic peptide and heparin could suppress ET-1 production [5,43]. The results of the current study indicate that RWDISQPY and captopril can reduce ET-1 secretion through inhibiting the activity of ACE or by protecting vascular endothelin cells from oxidative stress. Previous studies demonstrated that captopril and oligopeptides (like RWDISQPY) rich in hydrophobic amino acids could effectively protect the EA.hy926 cells from oxidative damage induced by hydrogen peroxide [5,30]. However, the action mechanism of RWDISQPY on ET-1 needs further study. Until now, reports referring to the effect of the peptides on intracellular endothelin-1 are limited. In the current study, RWDISQPY could effectively inhibit the activity of ACE and lower the intracellular ET-1 content, resulting in a decrease in blood pressure (Figure 3 and Figure 7). This may be one of the antihypertensive mechanisms of RWDISQPY. Now, some ACE-inhibitor drugs such as lisinopril, captopril and enalapril are widely used to lower blood pressure in clinical settings, but the noticeable side effects including hyperkalemia, decreased renal function and dry cough raise more concerns [2,31]. In contrast, antihypertensive peptides derived from natural sources without side effects have better prospects [4]. RWDISQPY, an antihypertensive peptide derived from *S. maclurei*, has considerable ACE-inhibitory activity and a potential mechanism of action similar to that of captopril and lisinopril, and shows potential applications in antihypertensive products or functional foods.

## 4. Conclusions

One novel ACE-inhibitory peptide, RWDISQPY, was identified in *Sargassum maclurei* protein hydrolysates. It demonstrated high ACE-inhibitory activity (72.24 μM) with a competitive mode of inhibition, and exerted a significant antihypertensive effect in SHRs. The results from the docking simulation suggest that RWDISQPY displays a strong binding power to the active pocket S1 and S2 of ACE via short hydrogen bonds. In general, it seemed to exert antihypertension through inhibiting the activity of ACE and suppressing the expression of ET-1, which was similar to the antihypertensive mechanism of captopril. However, the bioavailability and mechanism of action in vivo should be studied in future work.

## Figures and Tables

**Table 1:** Amino acid content of *S. maclurei* protein (g/100 g protein).

**Amino Acid**	**Thr**	**Leu**	**Ile**	**Val**	**Phe**	**Lys**	**Met**	**Tyr**	**Trp**
Content	3.62 ± 0.16	6.20 ± 0.38	3.75 ± 0.23	4.67 ± 0.22	4.05 ± 0.14	4.11 ± 0.12	1.29 ± 0.09	2.13 ± 0.17	2.33 ± 0.22
**Amino Acid**	**Asp**	**Glu**	**Cys**	**Ser**	**His**	**Gly**	**Pro**	**Ala**	**Arg**
Content	8.27 ± 0.06	29.77 ± 2.23	3.50 ± 0.43	3.32 ± 0.12	1.35 ± 0.08	4.26 ± 0.20	3.93 ± 0.62	7.92 ± 0.63	3.87 ± 0.12

**Table 2:** Predicted ACE-inhibitory activity and physicochemical properties identified in *S. maclurei* protein hydrolysates by LC–ESI–MS/MS and screening in silico.

Peptide	Theo. MH+ (Da)	B (μM)	ALC (%)	SVMS	Prediction	Hydrophobicity	Net Hydrogen	Calculated pI	Solvation	IC_50_ (μM) on ACE-Inhibitory Activity
RVLSAAFNTR	1134.42	ND	93	−1.19	Non-AHT	−0.24	1.20	12.01	0.28	ND
IMNILEK	860.16	0.0012	77	−0.46	Non-AHT	−0.02	0.71	6.35	0.88	ND
GGVQAIR	699.90	0.0333	71	−0.86	Non-AHT	−0.09	0.86	10.11	0.25	ND
KAALMEK	790.07	ND	75	−1.20	Non-AHT	−0.22	0.71	8.94	0.53	ND
GVFDGPCGT	852.05	ND	80	−0.43	Non-AHT	0.08	0.22	3.80	0.52	ND
SGVFDGPCGT	939.14	ND	81	−0.35	Non-AHT	0.04	0.30	3.80	0.47	ND
QNIGDPR	798.94	0.0016	77	−0.27	Non-AHT	−0.43	1.29	6.19	−0.15	ND
AYSSGVSFK	945.15	0.0044	73	−0.80	Non-AHT	−0.03	0.67	8.94	0.61	ND
RWDISQPY	1063.47	0.0016	91	0.34	AHT	−0.30	1.25	6.19	0.47	72.24
LVYIVQGR	947.25	0.0012	78	−0.74	Non-AHT	0.01	0.88	9.10	0.76	ND
KPGGSGR	657.82	0.0733	70	−0.10	Non-AHT	−0.39	1.00	11.01	−0.21	ND
LGLSAKNYGR	1078.36	0.0008	77	−0.68	Non-AHT	−0.21	1.00	10.01	0.28	ND
KEAWLIEK	1016.31	0.0411	90	−0.75	Non-AHT	−0.20	0.88	6.49	0.55	ND
REVADDK	831.96	0.0007	74	−0.02	Non-AHT	−0.59	1.29	4.56	−0.52	ND
ENFFFAGIDK	1187.44	ND	87	−0.69	Non-AHT	−0.01	0.60	4.38	0.63	ND
QEMVDK	748.93	ND	73	−0.10	Non-AHT	−0.39	1.00	4.38	0.19	ND
EEEEEEQQQ	1177.23	0.0372	79	−0.30	Non-AHT	−0.64	1.33	3.46	−0.58	ND

Abbreviations: B, the potential ACE-inhibitory activity calculated using published data in the BIOPEP databases; ALC, average local confidence; AHT, antihypertension; SVMS, vector machine software score. The physicochemical properties were theoretically calculated by the AHTPDB databases.

**Table 3:** Docking scores and hydrogen bonds observed between the peptide RWDISQPY and ACE from the molecular docking simulation.

Ligand	T-Score	Hydrogen Bonds Number	Distance (Å)
RWDISQPY	10.70	10	Glu384: 1.99; ASP415:2.08; His353:2.72; Tyr523:2.52; Thr282:1.82; 2.04; Gln281:2.04; Asn277:2.73; Lys511:2.44

## Supplementary Materials

The following are available online at https://www.mdpi.com/2072-6643/12/3/653/s1, Figure S1: HPLC analysis of peptide RWDISQPY on Kromasil 100-5 C_18_ column (4.6 × 250 mm); Figure S2: The MS/MS analysis of the identified peptide RWDISQPY; Figure S3: Effects of RWDISQPY on body weight (A) and heart rate (B) of spontaneous hypertensive rats (SHRs).
