# Supplementary material for: Efficacy of a Novel ACE-Inhibitory Peptide from Sargassum maclurei in Hypertension and Reduction of Intracellular Endothelin-1
Source: Nutrients. 2020 Feb 28;12(3):653. doi: 10.3390/nu12030653 (PMC7146574; doi:10.3390/nu12030653)
Supplement: Supplementary file 1 [file nutrients-12-00653-s001.pdf]

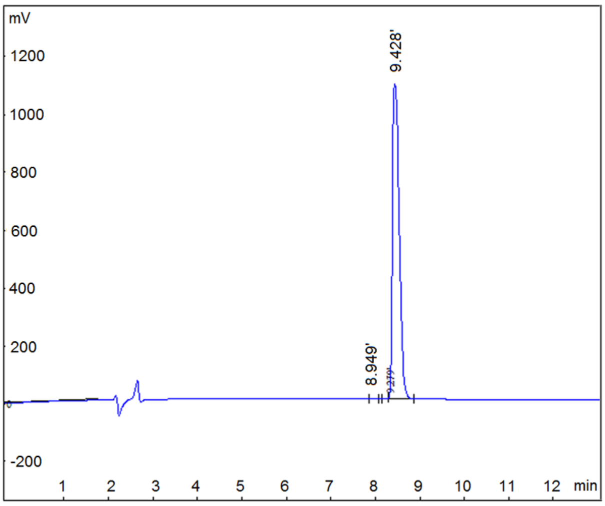

Figure 1. HPLC analysis of peptide RWDISQPY on Kromasil 100-5 C_{18} column (4.6 × 250 mm). Separation was performed at a flow rate of 2.2 mL/min and a linear gradient width of 20%-38% acetonitrile (containing 0.1% TFA) within 14 min.

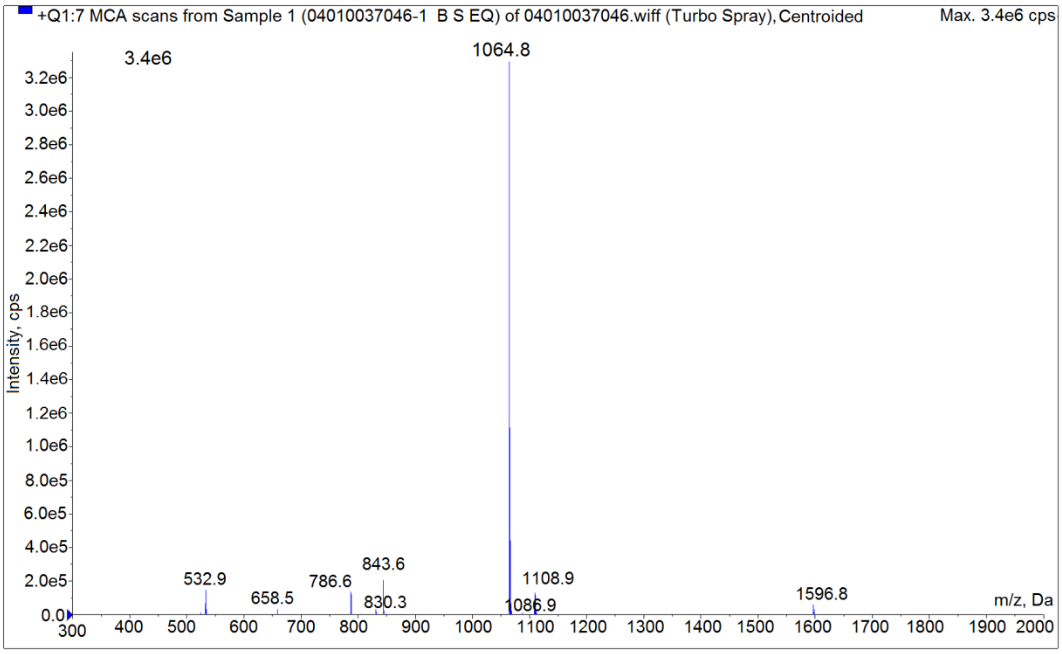

Figure 2. The MS/MS analysis of the identified peptide RWDISQPY.

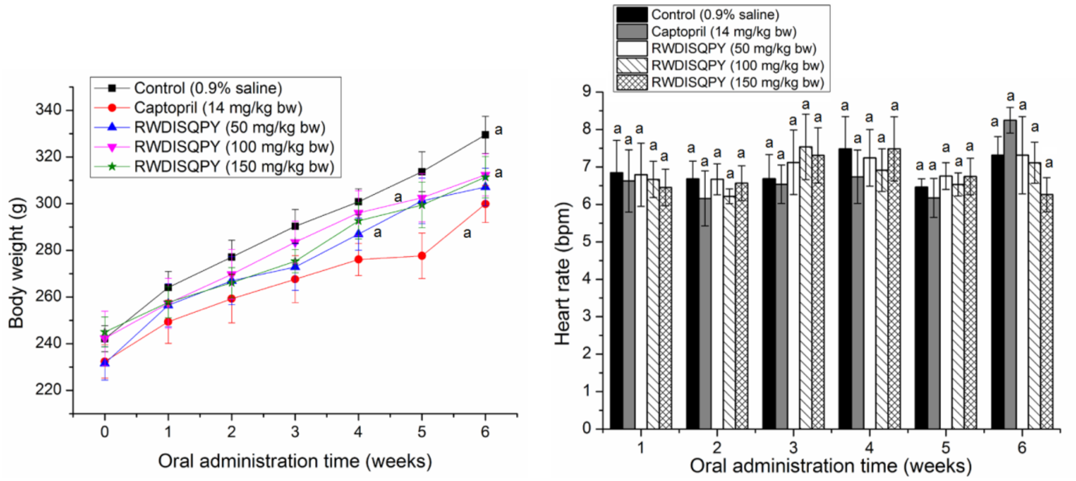

(A) **(B)**

Figure 3. Effects of oral administration of RWDISQPY on body weight **(A)** and heart rate **(B)** of spontaneous hypertensive rats (SHRs). SHRs in low-, middle- and high-dose groups were orally administered peptide at 50, 100 and 150 mg/kg/ body weight (bw) every day, respectively. SHRs of the positive control group were given captopril at 14 mg/kg/ body weight once daily, whereas the SHRs in the blank group were just given saline (0.5 mL). Different small letters above the bars or the near lines (a–c) mean significant difference ($p < 0.05$).
